# Application of the generalized shift operator to the Hankel transform

**DOI:** 10.1186/2193-1801-3-246

**Published:** 2014-05-14

**Authors:** Natalie Baddour

**Affiliations:** Department of Mechanical Engineering, University of Ottawa, Ottawa, Ontario K1N 6N5 Canada

## Abstract

It is well known that the Hankel transform possesses neither a shift-modulation nor a convolution-multiplication rule, both of which have found many uses when used with other integral transforms. In this paper, the generalized shift operator, as defined by Levitan, is applied to the Hankel transform. It is shown that under this generalized definition of shift, both convolution and shift theorems now apply to the Hankel transform. The operation of a generalized shift is compared to that of a simple shift via example.

## Introduction

It is well known that the Hankel transform does not satisfy a convolution or shift theorem in the simple way as that the Fourier and Laplace transforms (Piessens 2000), reducing its apparent utility. This follows because the Bessel functions do not possess a simple addition formula in the same way that the exponentials satisfy *e*^*i*(*x* + *y*)^ = *e*^*ix*^*e*^*iy*^. The operation of convolution between two functions consists of a shift in one of the functions, a multiplication with the other function, followed by an integration (or summation for discrete transforms) over all allowable shifts. Thus, the lack of a convolution theorem for the Hankel transform follows because of the lack of a simple expression for the shift of a function in the Hankel transform domain.

Levitan introduced the idea of a generalized displacement operator (Levitan 2002). As useful as this concept might be, to the best of the author’s knowledge it does not appear to have seen much application in the physics or engineering literature. In this paper, Levitan’s generalized displacement operator is made concrete via application to the Hankel transform. We show that this leads to shift and convolution rules for the Hankel transform. By way of several examples, the Hankel (generalized) shift is compared to the standard simple shift.

## Background

Several definitions of the Hankel transform appear in the literature. In this paper, we use the definition of the *n*th order Hankel transform as defined by Piessens in (Piessens 2000) to define the Hankel transform as1

where *J*_*n*_(*z*) is the *n*th order Bessel function. Here, *n* may be an arbitrary real or complex number. However, an integral transform needs to be invertible in order to be useful and this restricts the allowable values of *n*. If *n* is real and *n* > − 1/2, and under suitable conditions of integrability of the function, the transform is self-reciprocating and the inversion formula is given by2

Thus, the Hankel transforms takes a function *f*(*r*) in the spatial *r* domain and transforms it to a function *F*(*ρ*) in the frequency *ρ* domain. This relationship is denoted symbolically as *f*(*r*) ⇔ *F*(*ρ*).

## Generalized shift for the Hankel Transform

(Levitan 2002) introduced the idea of a generalized shift operator. Using Levitan’s definition, the generalized shift operator  indicates a shift of *r*_0_ acting on the function *f*(*r*) is defined by the formula (Levitan 2002).3

where *F*(*ρ*) is the Hankel transform of *f*(*r*) and we write the generalized-shifted function as , as a reminder that the shifted function is now a function of *r* and *r*_0_. The operator  acting on the function *f*(*r*) indicates a shift in *f*(*r*) by *r*_0_. The intuitive definition of the Hankel shift (generalized shift), as defined in (3), is that of the inverse Hankel transform of *F*(*ρ*)*J*_*n*_(*ρr*_0_), whereas the unshifted function would be the inverse Hankel transform of *F*(*ρ*) alone, without the multiplication by *J*_*n*_(*ρr*_0_). In essence, Equation (3) says that multiplication by *J*_*n*_(*ρr*_0_) in the Hankel domain is equivalent to a generalized shift in the spatial domain.

This definition follows the same expression used for the Fourier transform, that is:4

In (4), the star denotes the complex conjugate and follows the definition of generalized shift as given by Levitan. As previously pointed out, the simple shift, *f*(*t* − *t*_0_), follows from the definition because . For the Hankel transform with Bessel functions, no simple equivalent expression exists, but the general structure of the shift operation for the Fourier transform (left-hand side of Equation (4)) is the same.

It is noted that using the definition of Hankel transform given in (1), the shifted function  can also be written as5

where the order of integration has been reversed and where:6

The interpretation of the generalized shift operator from Equation (5) allows the shift to be seen directly as an operation on the original untransformed function. The definition as given in (3) allows for better physical interpretation of the definition (in particular in comparison with the familiar Fourier transforms) and also permits the simple proofs to follow for the shift and convolution rules.

## Generalized shift rule

In keeping with the standard shift-modulation rule of the Fourier transform, the definition of the Hankel generalized shift allows for a similar rule to be derived for the Hankel transform. The Hankel transform of the generalized-shifted function is given by application of Equation (1) to the generalized-shifted function in (3) as7

where the order of integration has been reversed. Using the orthogonality (closure) of the Bessel functions (Watson 1995; Abramowitz & Stegun 1964)8

it then follows that:9

In other words, a (generalized) shift in the spatial domain is equivalent to multiplication by *J*_*n*_(*ρr*_0_) in the Hankel domain or, if *f*(*r*) ⇔ *F*(*ρ*) then *f*(*r*|*r*_0_) ⇔ *F*(*ρ*)*J*_*n*_(*ρr*_0_). This follows the same rule as for the Fourier transform where the Fourier transform of *f*(*r* − *r*_0_) is given by .

## Modulation rule

Since the Hankel transform is self-reciprocal, a “modulation” rule similar to that of the Fourier transform can easily be derived. The Hankel transform of *J*_*n*_(*rρ*_0_)*f*(*r*) is given by10

where the definition of the shifted transform follows by replacing *r*, *r*_0_, *ρ*, *f* with *ρ*, *ρ*_0_, *r*, *F* in the definition (3) so that11

In other words, if *f*(*r*) ⇔ *F*(*ρ*) then it follows that .

## Convolution rule

With the definition of the generalized shift, we define the Hankel convolution of two functions as:12

Equation (12) is in keeping with the typical definition of a convolution in the radial domain, where the simple shift *f*(*r* − *r*_0_) has been replaced with the generalized shift *f*(*r*|*r*_0_). Furthermore, we use the notation * _*H*_ to denote that this is a Hankel convolution, meaning that the generalized Hankel shift operator is used instead of the simple shift operator. It is noted that other authors define a Hankel convolution without reference to the generalized shift operator. In all those cases, the integral of a triple product of Bessel functions is used to define the Hankel convolution, for example in (Tuan & Saigo 1995; Malgonde & Gaikawad 2001; de Sousa Pinto 1985; Belhadj & Betancor 2002). The mathematical properties of Hankel convolutions are analyzed in (Tuan & Saigo 1995; Malgonde & Gaikawad 2001; de Sousa Pinto 1985; Belhadj & Betancor 2002; Betancor & Marrero 1993; Betancor & Marrero 1995; Cholewinski & Haimo 1966).

We now proceed to find the Hankel transform of the Hankel convolution as defined in Equation (12):13

Interchanging the order of integration gives: it then follows that14

In the preceding Equation, (14), the definition of the Hankel transform of *g*(*r*) has been used, in addition to the orthogonality of the Bessel functions. Equation (14) clearly states that the Hankel transform of the Hankel convolution is the product of the Hankel transforms, again in parallel with the standard result of Fourier transforms. Furthermore, interchanging *g* and *f* in the proof would give the same result, therefore it also follows that (*g* * _*H*_*f*)(*r*) = (*f* * _*H*_*g*)(*r*) and that the Hankel convolution commutes. Therefore, we have that:15

## Multiplication rule

Since the Hankel transform is self-reciprocating, then interchanging the roles of *f*(*r*) and *g*(*r*) with their transforms *F*(*ρ*) and *G*(*ρ*) in the previous derivation gives the result that the Hankel transform of the product *f*(*r*) *g* (*r*) is the Hankel convolution of their transforms (*G* * _*H*_*F*)(*ρ*) = (*F* * _*H*_*G*)(*ρ*), so that:16

### Example

In this section, we apply the preceding definition to a commonly used function. The Boxcar (or gate) function is defined in Hankel frequency space as:17

The zeroth order inverse Hankel transform of the Boxcar function is given by:18

**Figure 1:**
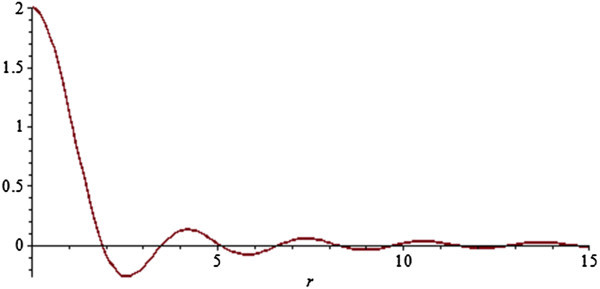
**Jinc function.**

**Figure 2:**
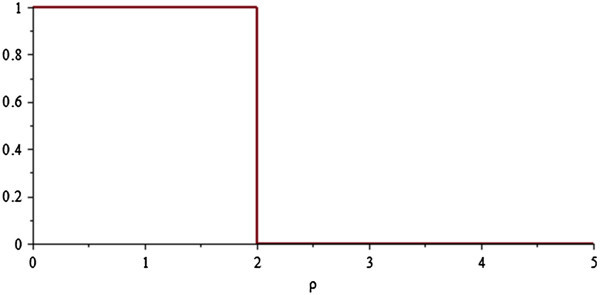
**Boxcar function** ***Π***_**2**_ ***(ρ)*** .

The function 2*J*_1_(*x*)/*x* is often termed the jinc or sombrero function and is the polar coordinate analog of the sinc function. Thus, we have that  are a Hankel transform pair. Plots of the jinc and its Hankel transform box car are shown in Figure 1 and Figure 2.

The generalized-shifted jinc function is thus given by Equation (3) and can be found in closed form via integrals given in (Watson 1995) as:19

**Figure 3:**
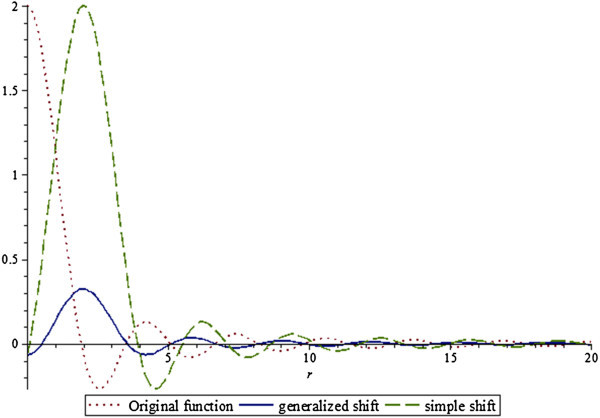
**Comparison of original function, generalized shift and simple shift, a = 2, b = 2.**

A comparison of the original jinc function [*a*/*rJ*_1_(*ar*)], its generalized shift *R*^*b*^[*a*/*rJ*_1_(*ar*)] and its simple shift  for *a* = 2 and *b* = 2 is given in Figure 3.

**Figure 4:**
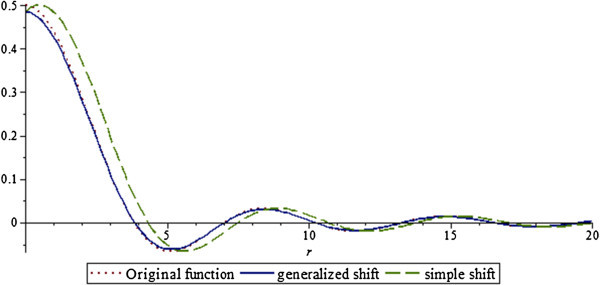
**Comparison of original function, generalized shift and simple shift, a = 1, b = 1/2.**

Clearly from Figure 3 (and it should be obvious from Equation (18)), the generalized and simple shift are quite different and it would be a mistake to think that one can be used in place of another for modeling and simulation purposes. A comparison of the original jinc function, its generalized shift, and its simple shift for *a* = 1 and *b* = 1/2 is given in Figure 4. For smaller values of the shift, the generalized and simple shifts are closer to each other.

## Discussion

The primary utility of the generalized shift function would appear to be that it is the function that permits the standard shift, modulation, multiplication and convolution rules to apply when using the Hankel transform.

We demonstrated in prior work (Baddour 2009; Baddour 2011) that the Hankel transform is part of a two-dimensional Fourier transform in polar coordinates and that taking a convolution over the radial coordinate only (a convolution using the simple shift and only over *r*) does not lead to any simplification because the process of shifting over the radial coordinate destroys radial symmetry. In fact, it was shown in (Baddour 2009; Baddour 2011) that a radially symmetric function shifted by  in polar coordinates is given by:20

Thus, for a function shifted along the radial axis (*θ*_0_ = 0), and supposing we are only interested in its values on the radial axis (*θ* = 0), then the simple shift in terms of the unshifted function is given exactly as:21

Generally speaking, the Hankel transform alone (without an accompanying angular coordinate Fourier transform to turn it into a two- dimensional Fourier transform) is most used in physical systems that have radial symmetry. Once the system is shifted in the radial direction - as is necessary to take a convolution - radial symmetry is lost and thus the proper, physically-meaningful transform would be a full 2D Fourier transform in polar coordinates. We showed in (Baddour 2009; Baddour 2011) that if a full (radial and angular) shift is taken in defining the convolution, then the 2D Fourier transform in polar coordinates does possess the standard shift, modulation, multiplication and convolution rules.

What we have demonstrated in this paper is that if it is desirable to use *only* the Hankel transform and work with only the radial coordinate, then the Hankel transform *does* possess the standard shift, modulation, multiplication and convolution rules – but only when the generalized definition of the shift is employed, not with the simple shift.

## Conclusions

We have shown that the Hankel transform *does* possess the standard shift, modulation, multiplication and convolution rules – but only when the generalized version of the shift is employed, not with the simple shift. We demonstrated by way of a simple example that the generalized shift and simple shift are not the same and thus not interchangeable for simulations of physical systems. The value of the generalized shift is that it permits the standard Fourier rules to apply to the Hankel transform. For the purposes of calculating physically meaningful convolutions, the simple shift in the radial coordinate should also be accompanied with an angular shift. A physically meaningful convolution implies integration with a shift over *all* physical coordinates allowed by the geometry of the problem. Fortunately, the full 2D Fourier transform in polar coordinates possesses the desired shift and convolution rules.
